# The Perceived Benefits of Digital Interventions for Behavioral Health: Qualitative Interview Study

**DOI:** 10.2196/34300

**Published:** 2022-03-30

**Authors:** Gabriela Marcu, Steven J Ondersma, Allison N Spiller, Brianna M Broderick, Reema Kadri, Lorraine R Buis

**Affiliations:** 1 School of Information University of Michigan Ann Arbor, MI United States; 2 Department of Obstetrics, Gynecology, & Reproductive Biology and the Division of Public Health Michigan State University East Lansing, MI United States; 3 Department of Family Medicine University of Michigan Ann Arbor, MI United States

**Keywords:** computers, mobile apps, screening, brief interventions, diagnosis, computer-assisted/methods, surveys and questionnaires, motivational interviewing, therapy, computer-assisted/methods, implementation, qualitative, mobile phone

## Abstract

**Background:**

Digital interventions have gained momentum in terms of behavioral health. However, owing to lacking standard approaches or tools for creating digital behavioral interventions, clinical researchers follow widely varying conceptions of how best to go about digital intervention development. Researchers also face significant cost-, time-, and expertise-related challenges in digital intervention development. Improving the availability of tools and guidance for researchers will require a thorough understanding of the motivations and needs of researchers seeking to create digital interventions.

**Objective:**

This study aims to understand the perceptions of behavioral researchers toward digital interventions, and inform the use of these interventions, by documenting the reasons why researchers are increasingly focusing their efforts on digital interventions and their perspectives on the perceived benefits that digital approaches can provide for researchers and intervention recipients.

**Methods:**

We conducted semistructured qualitative interviews with 18 researchers who had experience designing digital behavioral interventions or running studies with them. A convenience sample of interviewees was recruited from among users of the Computerized Intervention Authoring System platform, a web-based tool that facilitates the process of creating and deploying digital interventions in behavioral research. Interviews were conducted over teleconference between February and April 2020. Recordings from the interviews were transcribed and thematically analyzed by multiple coders.

**Results:**

Interviews were completed with 18 individuals and lasted between 24 and 65 (mean 46.9, SD 11.3) minutes. Interviewees were predominantly female (17/18, 94%) and represented different job roles, ranging from researcher to project or study staff. Four major themes came out of the interviews concerning the benefits of digital interventions for behavioral health: convenience and flexibility for interventionists and recipients, support for implementing evidence-based interventions with fidelity, scaling and improving access to interventions, and getting a foot in the door despite stigma and disenfranchisement.

**Conclusions:**

Interviewees described a number of important potential benefits of digital interventions, particularly with respect to scientific rigor, scalability, and overcoming barriers to reaching more people. There are complex considerations with regard to translating behavior change strategies into digital forms of delivery, and interventionists make individual, sometimes unexpected, choices with minimal evidence of their relative effectiveness. Future research should investigate how behavioral researchers can be supported in making these choices toward usability, ease of access, and approachability of digital interventions. Our study underscores the need for authoring platforms that can facilitate the process of creating and deploying digital interventions to reach their full potential for interventionists and recipients alike.

## Introduction

### Background

Digital behavioral interventions, which rely on digital technologies to promote behavior change and maintain health [1], have been growing rapidly over the past several decades. Digital approaches have unique potential benefits in promoting behavioral health, especially with regard to risky behaviors and sensitive or stigmatized topics (eg, drug and alcohol use [2-4], sexual health [5,6], and mood and anxiety [7]) that many are hesitant to discuss openly [8].

However, the development of such interventions requires significant expertise, planning, and financial resources [9-11]. Researchers interested in digital interventions may need more support in areas such as design guidance, software development, and financial or other logistical considerations. However, this support must be grounded in a deep understanding of the ways that investigators are using, or hope to use, digital interventions in their research. The purpose of this 2-part qualitative investigation is to better understand the motivations, needs, constraints, and experiences of researchers who have looked to digital interventions in their work addressing behavioral health. This study was conducted as part of a redesign of the Computerized Intervention Authoring System (CIAS) platform, a web-based authoring tool for researchers [12-14]. This study includes previous and current CIAS users and CIAS-naive users with interests in developing interventions for substance use [3,4], mental health [15], and HIV [16]. This convenience sample enabled us to investigate the experiences of researchers who have used an authoring tool to assist with the development, deployment, and scaling of digital interventions.

### Objectives

In this paper (part 1), we seek to document the reasons why these researchers used digital health interventions to address behavioral health. In a companion paper (part 2 [17]), we report common barriers to the intervention creators’ experience and key considerations that go into the design and implementation of digital interventions.

## Methods

### Overview and Ethics Approval

We conducted semistructured interviews with former, current, and potential future CIAS users, including investigators and research study staff. All methods used in this study were approved by the University of Michigan human subjects review board (HUM00171197) and Wayne State University institutional review board (IRB-19-10-1340) and were reported following the COREQ (Consolidated Criteria for Reporting Qualitative Studies) guidelines.

### CIAS Platform

CIAS is an internet-based intervention authoring tool that allows users to easily build, edit, and share web-based digital interventions without needing to have computer programming experience. The CIAS Health Insurance Portability and Accountability Act–compliant platform provides researchers with the ability to develop tailored and personalized text-based interventions that can be narrated by a variety of avatars, both human and animal, capable of multiple different actions and voices (supporting >40 different languages, with male and female versions of most, and a range of accents and dialects for some of the more commonly spoken languages). For researchers, CIAS supports screening functionality to determine study eligibility, in-platform randomization to multiple conditions, custom summary report generation, on-demand data access, SMS text messaging alerts flagging specified user inputs (eg, flags for suicidal ideation), instant language translation, a visual map showing intervention flow, and web analytics. CIAS supports intervention development features such as multiple question types, natural language reflections, branching and tailoring, and integration of video content. CIAS has been used by numerous research groups to develop a variety of digital interventions, many of which have focused on stigmatized behaviors such as substance use [15,18-21]. With support from the National Institutes of Health (EB028990), our team is redesigning CIAS from the ground up. This research was conducted as part of the redesign activities.

### Participant Recruitment

Interviewees were recruited via email solicitations from a purposive convenience sample of CIAS users via snowball sampling and through email to investigators familiar with conducting digital interventions. Most participants had experience with motivational interviewing (MI), an evidence-based technique to support behavior change [22], and in interventions on a variety of behavioral health topics. To be eligible to participate in this study, participants were required to be aged at least 18 years.

### Study Procedures and Data Collection

Owing to the COVID-19 pandemic, all interviews were conducted via teleconference and recorded for later transcription. Interviews were conducted in single sessions between February and April 2020 by 2 trained female study team members (BMB and ANS) with expertise in user experience design and methodology and with Master’s of Science in Information degrees. The 2 interviewers took field notes during the interviews, which typically lasted no more than 60 minutes. Study participants did not know the interviewers and were not given prior access to interview questions or guides; however, all participants knew they were being interviewed as part of CIAS redesign efforts. At the beginning of each interview, the interviewers read a standard script focused on information pertaining to the study, and the interviewees gave verbal consent before the interview. At the conclusion of the interviews, interviewees completed a brief electronic survey to assess their comfort with CIAS (if they had used it previously) and collect basic demographics. Participants were neither granted access to interview transcripts for review nor asked to comment on the findings. Interviewees were offered a US $20 check, which was mailed to their home, for each interview. Interviews were conducted until saturation was reached.

### Analysis

Analysis began with a debriefing between the 2 interviewers after each interview, during which they reviewed their individual notes and gradually synthesized data across interviews, looking for patterns. Interpretations of the data and identified patterns were discussed at meetings with the full study team every other week. This stage of analysis categorized participants within the common roles they played in their interactions with the CIAS or other digital interventions. Interview recordings were then transcribed, and inductive thematic analysis was conducted by 2 coders (BMB and ANS), including an initial round of full analysis that finalized a set of patterns that became the themes of this paper, followed by a second round of analysis that then validated these themes and confirmed connections among them. Memoing was used throughout this process.

## Results

### Overview

We solicited 24 former and current CIAS users for participation in this study. Of the 24 participants, the interviews were conducted with 17 (71% response rate). In addition, we solicited additional CIAS-naive users familiar with digital interventions through email recruitment using the University of Michigan Department of Family Medicine listserv and recruited 1 additional participant. In total, we completed interviews with 18 participants. The interview duration lasted between 24 and 65 minutes (mean 46.9, SD 11.3 minutes). Interviewees were predominantly female (17/18, 94%) and represented different job roles, ranging from investigator to project or study staff. Table 1 displays the aggregate interviewee characteristics, and Table 2 displays brief descriptions of each interviewee.

**Table 1:** Aggregate interviewee characteristics (N=18).

Characteristics		Values, n (%)
**Gender**		
	Male	1 (6)
	Female	17 (94)
**Job title (all that apply)**		
	Researcher	13 (72)
	Psychologist	4 (22)
	Other (project manager or coordinator and research assistant or associate)	5 (28)
**Employer**		
	Academic institution	15 (83)
	Foundation	1 (6)
	Contract research organization	1 (6)
	Other (nonprofit research organization)	1 (6)
**Race**		
	African American or Black	4 (22)
	White	13 (72)
	Prefer not to answer	1 (6)
**Ethnicity**		
	Hispanic or Latino	2 (11)
	Non-Hispanic or Non-Latino	16 (89)
**Education**		
	Bachelor’s	6 (33)
	Master’s	4 (22)
	Beyond a master’s degree	8 (44)
**Self-reported proficiency with CIAS^a^ (n=17)**		
	Novice	3 (18)
	Proficient	4 (24)
	Advanced	7 (41)
	Expert	3 (18)

^a^CIAS: Computerized Intervention Authoring System.

**Table 2:** Individual interviewee descriptions.

ID	Self-reported title	Employer type	Highest level of education obtained	Gender
P1	Researcher; psychologist	Academic institution	Beyond a master’s degree	Female
P2	Researcher	Foundation	Master’s degree	Female
P3	Project manager	Academic institution	Bachelor’s degree	Female
P4	Psychologist	Academic institution	Beyond a master’s degree	Female
P5	Project manager	Academic institution	Master’s degree	Female
P6	Researcher	Academic institution	Beyond a master’s degree	Male
P7	Researcher	Academic institution	Beyond a master’s degree	Female
P8	Researcher; psychologist	Academic institution	Beyond a master’s degree	Female
P9	Researcher; psychologist	Academic institution	Beyond a master’s degree	Female
P10	Researcher	Academic institution	Bachelor’s degree	Female
P11	Researcher	Academic institution	Bachelor’s degree	Female
P12	Research assistant	Academic institution	Bachelor’s degree	Female
P13	Researcher	Contract research organization	Master’s degree	Female
P14	Researcher; research project coordinator	Academic institution	Master’s degree	Female
P15	Researcher	Nonprofit research organization	Beyond a master’s degree	Female
P16	Research associate	Academic institution	Bachelor’s degree	Female
P17	Researcher	Academic institution	Bachelor’s degree	Female
P18	Researcher	Academic institution	Beyond a master’s degree	Female

### Benefits of Digital Interventions

#### Overview

Our interviews revealed the perceived benefits of digital interventions from the perspective of researchers focusing on interventions for behavioral health. Grounding our interviews in the use of the CIAS platform led to themes focused on the concrete, current, or near-future capabilities of digital interventions. Interviewees most commonly pointed to four categories of potential: increased convenience and flexibility, implementing evidence-based interventions with fidelity, scaling interventions and improving access, and getting a foot in the door despite stigma and disenfranchisement.

#### Convenience and Flexibility for Interventionists and Recipients

Researchers described looking to digital interventions as they allow for more flexibility in where and when they are delivered. Interviewees discussed the potential for the digital approach to increase convenience and flexibility for researchers and the populations they are looking to reach. One of the researchers noted that the convenience of accessing a digital intervention is what an increasing number of generations is coming to expect of any service they receive:

We have water, we have food, and we have cell phones. Everyone is on their cell phones. Even the older generations have a phone. So, I think that CIAS is very convenient...I would like to see it a component like telemedicine is now. Telemedicine has its own phone number on the back of newer insurance cards now...And I think that would definitely be rewarding to a lot of people. Because I see a lot of Millennials, and even the GenXers they’re gravitating towards computer-based help. They're not jumping into their cars, spending $3 for a gallon of gas to go to somebody’s clinic. They're hopping online.

P10

Evolving expectations about service delivery extended to the clinical point of view. Another researcher explained their view of how digital tools would likely support research and clinical visits in the future:

I think [CIAS] is going to allow us to deliver interventions at times that are more convenient for participants. As everything goes more mobile and more digital, being able to shoot a participant a link, enroll them in a study, shoot them a link, let them complete their follow up visits via that link, send me a text when they’re done, shoot them an e-incentive. I think that’s really going to change just delivering behavioral interventions in clinic and around clinic visits to delivering them, not when the client wants, so you’re still on timed intervals, but when it’s most convenient for the client.

P5

However, researchers most often cited benefits for the recipients, including more choice in how they interact with the content. Recipients can complete intervention sessions wherever they are most comfortable and during a time they deem most appropriate while still staying within the allotted timeline of the intervention. This increase in flexibility may heighten the appeal of behavioral interventions to recipients, and researchers speculated that this could help with engagement:

I think a mobile intervention delivered on a computer is less threatening, and on the surface seems easier or more palatable to a research participant. “I don’t have to go anywhere to do this, it can come to me at a time that’s convenient to me”...The thought of coming into the office to do psychotherapy sessions is unappealing for many. But, looking at the convenience of my home at a time that is convenient for me, it may be more appealing.

P7

Researchers were especially attuned to the needs of recipients who experienced symptoms such as fatigue, which could affect their engagement with an intervention. In these situations, interviewees had the sense that delivering interventions remotely via computers was helpful:

As far as it being remote, our participants, actually, the ones that do follow through and do their things, they love it because we’re measuring their level of fatigue, so we aren’t bothering them with making them come somewhere to have their visit completed. They can do it when they feel ready, feel able so it being remote is actually getting people to stay on, it’s easier to get them to stay on because they don’t have to go anywhere.

P11

As we will discuss in following sections, the convenience of recipients accessing interventions from their homes is also important when addressing mental health and other sensitive or stigmatized topics.

#### Implementing Evidence-Based Interventions With Fidelity

Digital interventions also have the benefit of providing a structure that can support the use of evidence-based strategies with fidelity—that is, as intended or designed to ensure efficacy. Many of the researchers we interviewed were experienced in using MI and reflected on how and why they used digital interventions in concert with this approach. Digital interventions control the structure of how recipients experience the intervention, and researchers also remarked on how easily they could monitor the intervention and collect various kinds of data as recipients engage with it:

It’s all programmed so there’s less issues with fidelity and fidelity monitoring and so I guess that’s the piece that changes CIAS, problem solving and/or developing strategies to monitor and track intervention fidelity. We still have to track delivery, so we still have to figure out “did they get it? did they complete it?” but we’re not dealing with a human counselor who can kind of not follow the roadmap.

P7

The analogy of an intervention as a road map suggests that systematic mapping of a digital intervention’s user experience can serve to ossify the intervention and standardize how it is delivered to recipients. This mapping is performed once when the digital intervention is designed, and then researchers know that it will be delivered consistently each time. Therefore, interviewees indicated that improvements in fidelity could come about not least by reducing reliance on humans, each of whom must be trained to effectively deliver the intervention:

Home visitors were mostly...they’re not trained clinicians...they don’t necessarily have the skills to really implement a brief motivational intervention in a way that it would be effective...so we sort of landed on CIAS where it’s electronic and would be fully implemented electronically so the home visitor doesn't...have to learn how to do brief interventions.

P15

Others similarly described how they had used digital interventions to overcome the variability inherent in training humans to deliver interventions, such as MI:

Most of the literature says that no matter how well you train people, that they don't deliver MI with high fidelity to the components that are supposed to be there at all. So, you spent all these hours and hours training people, and they still don't do it the way it's intended. So, of course, you know, having, and always doing it the same way makes a huge difference.

P1

In addition to reliance on humans to deliver interventions consistently, researchers described challenges with training against the natural tendency to provide advice. By contrast, digital interventions were seen by interviewees as nonjudgmentally enabling a recipient to reflect on their health behaviors without the potential intrusion of another person’s thoughts:

Our biggest challenge when teaching navigators...is getting them to not move into the role of “here's how to fix this and giving advice prematurely". And kind of having their own agenda. So, I think the pieces that can be done really well with CIAS are, you know, non-judgmental opportunities for a person to kind of look at their situation.

P9

Interestingly, researchers saw an advantage in the fact that more nuanced aspects of MI, such as empathy, cannot be easily conveyed through a digital intervention. As recipients have lower expectations for warmth and understanding than they do of a human, a digital intervention may not need to meet many requirements for an effective interaction:

You can get away with a lot more on a computer-based intervention than you can with an in-person intervention. So, for instance, the idea of empathy and understanding, and warmth, and avoiding advice-giving and stuff like that, there are issues within person interaction. I don’t think a lot of those are really problems with computer-based interventions. So, a computer can say “hey, wake up, go for a walk today” but if you told that to your roommate or boyfriend, that would be obnoxious, right? So, I wouldn’t say that thinking is different about in-person than they are with online interventions, but the capacity of the two to kind of do the “dance” differently.

P6

Other interviewees alluded to this dance between the intervention and the recipient, describing how they balance the advantages and disadvantages of digital interventions to implement evidence-based strategies. For example, when using MI, there is a trade-off between the nuance a human is capable of and the difficulty in achieving consistent delivery of the intervention. Some researchers choose to sacrifice some of the former to improve the latter with a digital intervention:

The role of the avatar in this [intervention]...I think it isn’t as good as a really good MI therapist delivering the intervention [using core principles like affirmations, empathy, non-judgement, no premature advice]. No, it’s not, but as someone who trains people on a regular basis, you know the things that CIAS can do and do consistently, are things that I struggle to train caseworkers and patient navigators to be able to do. And I think in that sense it’s a, it’s a consistent step forward and an improvement...CIAS just lets you sort of balance that out [any variation in individual skill or technique] and make sure that everybody’s getting some basic level of MI consistently.

P9

Therefore, one of the key reasons that our interviewees chose to adopt digital interventions was to improve the fidelity of their evidence-based interventions through consistent delivery, monitoring, and avoidance of factors that are very difficult to manage when training humans. On the other hand, the downside of increasing structure and fidelity with digital interventions is losing the human ability to pick up on cues from the recipient, have more open-ended conversations, and adjust the intervention in response.

#### The Way of the Future: Scaling Interventions and Improving Access

Researchers were enthusiastic about the potential for scaling to broader populations with digital interventions in the future. This argument was typically framed from a population health perspective, as a relatively small change in behavior can have significant consequences if it can be achieved widely:

If you can reach more people and have a small change, that’s important in shifting population behavior. So, digital mobile health interventions are the way of the future.

P7

Similarly, researchers described the benefits to public health efforts if apps could bring interventions to those who are most difficult to reach:

I think it would be hugely impactful. I think a lot of the issues, or a lot of the barriers that public health faces as an industry is kind of getting people involved and actual...a lot of the issues with public health is low income and low resources for a lot of communities, and providing something so easy to use and so easy to get as an app, and allow public health professionals to communicate with so many people much easier and much, much more can make a world of difference. Program building and eliminating disease, and you know providing a more healthy lifestyle for people and kind of guiding them through that process.

P17

One of the researchers further summarized the multilayered social factors that have resulted in populations being neglected and saw digital technologies as a way of addressing inequity:

There's this void within our community that a population is being ignored. A population that could find themselves in a mental health crisis. And they may be wheelchair bound. They may not have transportation. They may not have 24-7 care. So, a lot of the day-to-day operations of their life are online and on the phone. And it also fulfills a common item in our life.

P10

The level of access a person has to services can also change significantly during a crisis in their personal life or their environment. In such situations, digital interventions can be more accessible and immediately available to provide tangible help if a person cannot leave their home during a crisis, as demonstrated by the global pandemic:

CIAS is catching up with the times and so to speak, providing interventions that are needed that are immediate. But in a convenient way. Like, for example, right now CIAS is needed because we're quarantined to our home [due to the COVID-19 pandemic]. Let's say for example, a person is on the verge of a break. They're in an emergency mental health crisis, they can't leave their home for whatever reason that may be. So, they can access the phone, get on the phone, and talk to somebody with real information with real resources.

P10

With the ease of providing people access to interventions digitally, researchers highlighted the potential for scaling interventions as well as tailoring them to different people’s needs:

[Digital interventions are] really scalable, you can tailor it in an infinite number of ways.

P6

For example, recipients’ literacy may be a consideration, in which case digital interventions could engage the recipients through audiovisual interaction:

I think illiteracy is an issue for some of these folks. So, I think having a survey they could participate in where they didn’t have to worry about having to read things was something [important to us].

P9

The ability to reach people in new ways expanded researchers’ thinking about new kinds of interventions. More than just translating existing interventions into digital formats, they were excited to think innovatively based on novel possibilities now afforded to them:

[CIAS] definitely broadened my ideas—like what is possible...like how many people could be reached by this. I think that especially—I am from rural Alabama and there’s no way that you’re ever going to hire like a—even a social worker is a stretch—but to get someone into a clinic setting who is trained and qualified to do these [assessments] is a stretch. And, you know, having an option to give someone a tablet while they’re sitting there and they go through maybe a very quick screener and then they’re eligible for something. Or they’re at risk for something and you’re handing them something that’s interactive and personalized and can give them feedback without having to have another person on the other end, I think is genius and could help a lot of people.

P16

Some interviewees highlighted the potential for scaling access to interventions as the key reason they chose to use digital interventions:

And so, across all of those [potential benefits] for me the selling point has been getting interventions to more people more effectively.

P9

However, we note that this potential remained largely aspirational for many researchers in our study, an indication of ongoing challenges with the development of digital interventions.

#### Getting a Foot in the Door Despite Stigma and Disenfranchisement

Access to health interventions is even more challenging when targeting behaviors that are stigmatized or populations who may not be comfortable seeking help. Disclosure of certain behaviors or circumstances is a significant barrier to connecting an individual with appropriate support. Several researchers mentioned that digital interventions enabled them to broach the taboo topic of substance use among pregnant and postpartum women. Therefore, by facilitating disclosure of sensitive issues such as substance use or domestic violence, digital interventions may open the possibility of intervening:

Being able to deliver an intervention online...you can send someone a link and they can go through a program on their computer, that could potentially really help them without having to disclose to a doctor or social worker or research assistant. I think that has massive implications for being able to get people help that’s tailored to them. For substance use, I think it will help a lot...it has the potential in any kind of stigmatized, sensitive area, like in the doctor’s office - a domestic violence screener. In any kind of healthcare setting trying to screen for any type of sensitive issue, I think it has a lot of potential to help in those areas.[P13]

Notably, disclosure was brought up in the context of both clinic and home visits. Researchers were cognizant of protecting confidentiality, whether engaging someone in a public clinical setting or via a visitor to their home. A digital intervention, even if handed over to the recipient by a human, was seen as potentially protective of their confidentiality:

We’re hopeful that home visiting clients will be able to answer [sensitive] questions via an iPad, if you take away the idea that somebody else is judging you.

P2

Researchers described intentionally deploying digital interventions to convey to recipients the confidentiality of any disclosures:

The client doesn’t have to disclose if they don’t want to, and that’s a lot of the adapting that we’re doing is around that - making the client understand that this is confidential, that the home visitor doesn't need to know what they’re putting into the program.

P15

Barriers continue beyond screening and throughout the intervention process. Even if a potential intervention recipient can be identified (eg, through their disclosure) and linked to appropriate resources, they must be ready to accept information related to their health behavior. Therefore, the anonymity that digital interventions can afford may be helpful to an intervention itself getting a foot in the door, as one of the researchers explained within the context of engaging pregnant or postpartum individuals about their substance use:

These types of interventions, anonymous [and] in this population, do have the potential to get information and help to people who would not normally receive it because they would be too afraid to ask for it. I mean, just getting a foot in the door with these people and being able to give them psycho-education or resources at all, is a win and can really help people.

P13

Interviewees also described using digital interventions with populations they would not have expected to reach in the past, for example, because of racial disparities or the stigma against seeking mental health services:

We’re tapping into areas that we would have never thought of before. Like in terms of the disenfranchised populations [including based on their race], people who are afraid of being seen in the daylight entering into a mental health clinic. By having a phone, by having CIAS, we are getting them the help they need and we're giving them privacy...I do think we provide a comfort...I think our research becomes more and more validated because we're showing how flexible psychology and behavioral health can be.

P10

Once an individual is engaged in an intervention, the relative neutrality of an app may enable them to dig deeper into a topic, less impeded by impression management in the presence of another human. One of the researchers working on substance use among new mothers was hopeful that switching to anonymous engagement with a digital intervention would help recipients feel more comfortable disclosing sensitive information about their behavior:

The most important thing is that CIAS allows us to have confidentiality and allows the mothers to go through the interventions anonymously. It has removed the barrier, because the intervention before...the home visitor had to go down and take the mother through it and they found that almost zero of the mothers were truthful because they didn't like talking to a person about this. So, having our little parrot narrator [avatar] bring them through it will hopefully lead to us getting accurate data for once.

P13

As this researcher explained the ability of digital interventions to engage recipients differently, they also described the efficient progression possible by asking about certain health behaviors to connect the recipient with appropriate resources in interactive and tailored ways:

In an in-person setting in this kind of thing, you can almost never get a straight answer if you ask somebody like “We saw that you just had a baby. Care to talk about your substance use while you were pregnant?” Like no, no one’s going to say anything about that, so you always have to come from a perspective of...“in the month before you became pregnant, what was your substance use like?” But in an anonymous format, we can kind of work around those proxy questions and get more to the meat of it, like “how are you feeling about your substance use?” “Do you want information on how you can change?” and then if so, it immediately pops up, like here’s your list of resources for your town. [A digital intervention] definitely changes the way that you can ask questions.

P13

At the most basic level, interviewees acknowledged that intervention recipients might not even be receptive to the information provided by the intervention. In addition to removing the human interventionist and providing more privacy, digital interventions could address this issue by tailoring the information, how it is delivered, and when it is delivered:

I think one thing that CIAS has shown me is that people do appreciate the opportunity to learn information in private. And being that we thought they were absorbing or listening to what was being delivered from a person, they actually aren't. Because when you validate the information that they should have got from a person, electronically you see that it doesn’t line up. I think CIAS has given me a new respect for being able to deliver information in a way people will receive it...since we’re using MI, one thing that's pretty cool is that it does assess how much a person is willing to hear and it only gives them that and people tend to be interested in following up because they weren’t pressured into hearing something they didn’t want to hear and the time they didn’t want to hear it.

P5

Some interviewees reported that digital interventions had shown marked improvement in the experiences of both researchers and intervention recipients:

We really have found CIAS to be preferable to the alternatives of both live person interview and certainly compared to survey questions without having the avatar. It just keeps people’s attention and tends to, in my experience, you know, continue to engage them in a way that they’re hopefully answering the questions more validly.

P9

Such reports suggest that researchers have succeeded in getting their foot in the door with digital interventions, potentially even being better positioned to facilitate behavior change compared with other types of delivery. However, the use of digital interventions is still new, and research on their impact is limited.

## Discussion

### Principal Findings

We sought to better understand the reasons why some researchers who study topics related to behavioral health use digital interventions for lifestyle and behavior change interventions. The benefits of digital interventions that we identified among interviewees were 4-fold and included factors such as increased convenience and flexibility, fidelity to protocols, potential scalability and increased access to interventions, and the ability to reach individuals who may otherwise be unwilling to engage on matters of a sensitive nature.

### Increased Convenience and Flexibility

Interviewees consistently noted that digital interventions increased convenience and flexibility for both researchers and intervention recipients. For researchers, digital interventions are set up in advance before study recruitment, and they typically run automatically without much staff involvement and oversight. For intervention recipients, depending on how digital interventions are delivered, there is potential to engage at a time and place that is convenient. Especially for sensitive issues, intervention recipients may not wish to engage in public settings and with research or clinic staff with whom they are unfamiliar.

### Implementing Evidence-Based Interventions With Fidelity

#### Overview

The second theme that emerged from our interviews was the notion of fidelity to intervention protocols, both in terms of content and protocol fidelity. The algorithmic and automated nature of digital interventions can facilitate the delivery of content that is on schedule and consistently delivered in the same manner across intervention recipients. This protocol-based fidelity is more difficult with human interventionists.

#### Content Fidelity

Digital health interventions have the potential to ensure that content is delivered in the same way to all users, that is, in a nonjudgmental manner, which has been previously noted as a concern [23]. By digitally delivering content, we may be able to reduce social desirability bias. Moreover, automated interventions can finely tailor messages on a granular level in a way that is difficult for human interventionists.

#### Protocol Fidelity

The use of digital interventions can be tracked objectively and in detail. For example, log files and time stamps can verify when content is sent and when intervention recipients are engaged with the intervention. We can also objectively monitor measures of engagement, such as the number of times the content is accessed and the time spent engaging with content. Digital interventions allow information to be presented to recipients in a systematic way, which can allow for easier replication across various studies and with different populations, which could have a positive impact on the validity of a research study. This consistency can greatly benefit interventions that are based on validated therapeutic techniques, such as MI, by standardizing its delivery across all recipients.

#### Fidelity Is Not Guaranteed

However, it should be noted that the benefits of content and protocol fidelity may not always be realized. Experienced researchers know that new threats to fidelity that are otherwise absent from face-to-face interventions exist in a digital world. Digital interventions often cannot guarantee that automated digital content is received by the correct person or that users engage with the content in the intended manner. Although technical logs can tell us that messages were sent and the amount of time spent within interfaces or on specific pages, researchers cannot guarantee that content was received or read, that the content reached the correct users, or that users attended to the content. Technical issues can also reduce the actual exposure to intervention content. Researchers new to digital interventions may not understand that routine and robust monitoring of the back end system status is an important requirement of digital interventions. Waiting on intervention recipients to report that an intervention is not properly working can lead to missed data collection and poor intervention fidelity. Being prepared to respond to system failures in a prompt fashion can reduce these potential issues. In their review of digital interventions adapting MI, Shingleton and Palfai [24] found that fidelity measures were often not reported in the literature.

### Scaling Interventions and Improving Access

The third theme that emerged from our interviews was the unique potential of digital interventions to scale up and improve access to recipients. Regarding scalability, although initial costs are not insignificant for digital interventions, there is evidence that they can be cost-effective once developed [25]. However, robust data on digital interventions and cost-effectiveness are lacking [4,26-29]. From a research perspective, despite the high initial costs, digital interventions may reduce the need for costly human interventionists in research, which shifts human capital needs to technology development needs and may affect how money in research budgets is allocated. This increased potential for scalability means that population-based interventions are within better reach, and even small improvements in individual health outcomes can make big population-level improvements.

Issues related to access were especially important to the interviewees in this study. As noted earlier, digital interventions are potentially available at any time and any place. Moreover, as smartphone adoption is so ubiquitous, mobile interventions, or web-based interventions optimized for mobile devices, have the potential to reach even greater numbers than more traditional desktop-oriented programs. Although digital divide issues still persist as individuals with a lower socioeconomic status lag behind in terms of smartphone adoption, these populations with lower socioeconomic status tend to have better access to the internet via mobile phones than through computers and broadband in the home [30], which makes digital health interventions more important for reaching these populations. Digital interventions also have the potential to reach groups of people who may be hard to reach, such as those with physical disabilities, insufficient transportation, or significant time constraints. The nature of digital interventions may also increase the potential to reach small, distributed groups of people who may have unique needs that could be too costly to support in person, such as individuals with rare diseases.

Beyond the potential to reach a greater number of people, digital interventions also have the potential to reach users in moments where they may be most efficacious [31]. For example, just-in-time adaptive interventions provide interventions at times where they may be most useful and at escalating doses that may lead to better outcomes [32]. Although most research in this area has been conducted with smoking cessation and physical activity and weight management interventions, just-in-time adaptive interventions have been previously used to intervene in sensitive behaviors such as risky substance use among adolescents and emerging adults [33]. Screening, Brief Intervention, and Referral to Treatment (SBIRT) interventions have also been used to intervene with intervention recipients at times when they may be available and receptive to interventions, such as before clinical encounters with primary care physicians or immediately following training sessions, and have been focused on topics such as substance use, intimate partner violence, and mental health [34]. These increases in convenience and flexibility for intervention recipients may yield downstream benefits to researchers and potentially translate to increased intervention engagement and completion, which may also yield positive improvements in health outcomes.

### Getting a Foot in the Door Despite Stigma and Disenfranchisement

Findings from this study revealed that one perceived benefit of digital interventions for mental health and other stigmatized areas and behaviors is the opportunity to reach people concerning delicate topics when they are ready for and receptive to engaging in them. The anonymity provided by digital interventions may allow recipients to feel more comfortable disclosing sensitive information and engaging with related content. This may also afford users greater opportunities to delve into topics that they find more difficult to talk about. It is possible that this might lead to a greater level of honesty that could contribute to more successful intervention outcomes. Indeed, prior work has shown that self-reporting through digital formats can promote greater self-disclosure of undesirable behaviors and sensitive issues [8].

### Future Directions

Given the range of potential advantages our interviewees described and the optimism and early gains they expressed, research is needed to understand the impact of delivering interventions digitally. For example, research should investigate the user experience from each stakeholder’s point of view, identify potential unintended consequences, compare various digital and nondigital modalities for delivering interventions, and understand the disadvantages of digital delivery and how they can be addressed through careful intervention design and implementation. As mentioned in previous sections, research is needed to better understand the ways in which human-human interaction differs from human-computer interaction in terms of key factors such as perceived empathy, understanding, and friendliness.

### Limitations

This study had several limitations that must be highlighted. This study was conducted as part of the redesign activities for CIAS, and therefore, it does not represent all researchers who use digital interventions for behavioral health. Moreover, our small sample size may have affected generalizability. As our convenience sample of researchers comprised CIAS users and those familiar with our redesign efforts, they may have felt compelled to aid our study activities, which could have introduced both selection bias and response bias. Finally, our focus on CIAS users meant that we only spoke to those individuals who were responsible for creating and managing digital interventions. Although many of our interviewees have conducted qualitative work with their own recipients to understand their perspectives, we did not speak directly with recipients of digital interventions. Our findings are limited to the perceptions of digital intervention creators and study teams.

### Conclusions

Digital interventions are seen by interventionists as having many benefits for both interventionists and recipients, such as greater flexibility in how, where, and when an intervention is delivered; potentially improving the fidelity of and access to interventions; and overcoming challenges of reaching populations despite stigma and disenfranchisement. More research is needed to measure the actual benefits of digital interventions and better understand whether they align with researcher expectations and hopes. A comparison of perceived and actual benefits may also inform better support for creating digital behavioral interventions that more effectively meet their potential.

## Abbreviations

CIAS: Computerized Intervention Authoring System
COREQ: Consolidated Criteria for Reporting Qualitative Studies
MI: motivational interviewing
